# Nanometer resolution optical coherence tomography using broad bandwidth XUV and soft x-ray radiation

**DOI:** 10.1038/srep20658

**Published:** 2016-02-10

**Authors:** Silvio Fuchs, Christian Rödel, Alexander Blinne, Ulf Zastrau, Martin Wünsche, Vinzenz Hilbert, Leif Glaser, Jens Viefhaus, Eugene Frumker, Paul Corkum, Eckhart Förster, Gerhard G. Paulus

**Affiliations:** 1 Institute of Optics and Quantum Electronics, Friedrich-Schiller-University Jena, Jena, Germany; 2 Helmholtz-Institute Jena, Jena, Germany; 3 SLAC National Accelerator Laboratory, Menlo Park, United States; 4 Deutsches Elektronen-Synchrotron DESY, Hamburg, Germany; 5 Joint Attosecond Science Lab, National Research Council of Canada, Ottawa, Canada

## Abstract

Optical coherence tomography (OCT) is a non-invasive technique for cross-sectional imaging. It is particularly advantageous for applications where conventional microscopy is not able to image deeper layers of samples in a reasonable time, e.g. in fast moving, deeper lying structures. However, at infrared and optical wavelengths, which are commonly used, the axial resolution of OCT is limited to about 1 *μ*m, even if the bandwidth of the light covers a wide spectral range. Here, we present extreme ultraviolet coherence tomography (XCT) and thus introduce a new technique for non-invasive cross-sectional imaging of nanometer structures. XCT exploits the nanometerscale coherence lengths corresponding to the spectral transmission windows of, e.g., silicon samples. The axial resolution of coherence tomography is thus improved from micrometers to a few nanometers. Tomographic imaging with an axial resolution better than 18 nm is demonstrated for layer-type nanostructures buried in a silicon substrate. Using wavelengths in the water transmission window, nanometer-scale layers of platinum are retrieved with a resolution better than 8 nm. XCT as a nondestructive method for sub-surface tomographic imaging holds promise for several applications in semiconductor metrology and imaging in the water window.

One of the remarkable properties of OCT^1^,^2^,^3^ is that the axial resolution solely relies on the brevity of the coherence lengths *l*_*c*_, which is determined by the bandwidth Δ*λ* of the light source. OCT has therefore become the standard technique in ophthalmology for investigating the retina *in vivo*^4^. Furthermore, OCT has many applications in metrology and in material characterization, e.g., inspection of LEDs^5^, solar cells^6^, or even the conservation of cultural heritage objects^7^. Technically, OCT is realized by splitting the light beam into a probe beam that is scattered at the sample under investigation, and a reference beam that is superimposed with the scattered light from the sample. Axial resolution is achieved by detecting the respective interferences as a function of the delay between two arms of an interferometer. Accordingly, cross-sections in different depths of the sample can be retrieved by changing the length of the reference arm (time-domain OCT). Scanning the reference arm length can in fact be avoided if the interference is recorded in spectral domain instead (frequency-domain OCT)^3^,^8^. The lateral resolution of OCT is achieved by conventional imaging.

Note that, in contrast to conventional imaging, the axial resolution is virtually not limited by beam properties such as the Rayleigh length. Rather, as evident from the above description, the axial resolution of OCT is given by the coherence length 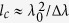 of the used radiation. Here, *λ*_0_ is the central wavelength of the radiation with the bandwidth Δ*λ*. Thus, the axial resolution of conventional OCT at infrared and optical wavelengths can reach about 1 *μ*m 2.

A self-evident way to improve the resolution further is the reduction of the central wavelength. The development of extremely broadband XUV and soft-X-ray sources in the framework of attosecond pulse generation^9^,^10^ and other physics disciplines in the last years suggests a realization of OCT in the XUV regime such that coherence lengths of a few nanometers are in reach. This variant of OCT is referred to as XCT^11^,^12^. However, XCT is limited in its applications by transmission windows of the host material of the sample owing to the strong photo-absorption of XUV radiation. We identified two scenarios of importance^11^. First, the transmission window of silicon at 40 to 12 nm gives rise to a resolution of 11 nm having a broad range of potential applications in semiconductor metrology. Second, the water transmission window of soft x-ray radiation from 4.4 (carbon absorption edge) to 2.3 nm (oxygen absorption edge) holds promise for a resolution of up to 3 nm. On the other hand, XCT has profound challenges. Besides absorption and XUV optics, the comparatively high dispersion in the XUV, which causes varying optical path lengths for different wavelengths in the sample, has to be taken into account in order to exploit the full potential of XCT.

In this letter, we present the first demonstration of XCT showing that layered nanometer-sized systems can be investigated non-invasively. The setup utilizes broadband XUV radiation, a soft x-ray spectrometer and samples with nanometer-scale layers buried in different host materials. The retrieved cross-sectional images have depth resolutions better than 18 nm in the silicon window and 8 nm in the water window, respectively, which are close to the theoretical resolution described above. A three-dimensional XCT image recorded via two-dimensional scanning of a layered sample system is shown in Fig. 1.

We reduced the technical complexity of OCT in the XUV spectral range as far as possible by using a variant of frequency-domain OCT shown in Fig. 2. Since XUV light is completely absorbed within a few micrometers of propagation in air^13^, the sample and the optical parts are prepared and aligned in vacuum at an XUV beamline. We have chosen synchrotron radiation for demonstrating XCT owing to the high and stable photon flux and adjustable wavelength range (see Methods Section and Supplementary Notes online).

First, the XUV beam was focused on the sample under investigation using reflective optics. We implemented a spatial filter using an aperture at an intermediate focus such that spot sizes of 20–100 *μ*m can be achieved. This provides a good compromise with respect to reasonable lateral resolution and a high photon flux. An improvement of the lateral spot size is technically demanding but straightforward using diffraction-limited XUV beams and high-quality XUV optics. In the next step, the XUV radiation scattered from the sample is recorded with an efficient XUV spectrometer consisting of a 1000 lines/mm freestanding thin transmissive gold bar grating and a back-illuminated XUV-CCD with 2048 × 512 pixels and 13.5 *μ*m pixel size^14^,^15^. Alternatively, the wavelength of the XUV source is swept so that an XUV photo diode can replace the spectrometer.

Our XCT setup is a variant of common-path OCT where the sample arm and the reference arm share the same path^16^. This has been realized by a highly reflective capping gold or platinum layer on top of the sample, which provides the reference beam. The reflected waves of the capping layer and the scattered light from nanostructures inside the sample interfere and induce spectral modulations. This has the particular advantage that the reference and the sample arm are mechanically linked and the interferogram is insensitive to mechanical vibrations. In this work, we completely avoided the use of a freestanding broadband beam splitter as it is typically used in OCT because it is technically extremely demanding (see also Supplementary Notes online).

For understanding our variant of XCT, let us first consider a single layer buried at a distance *z*_*B*_. With the surface reflection in addition, this layer system can be regarded as a Fabry-Pérot interferometer (FPI). When this FPI is irradiated with broad bandwidth XUV radiation, the reflected intensity spectrum *I*(*E*), where *E* is the photon energy, exhibits a well-defined modulation frequency *ν*_*E*_. In the simple case of vertical irradiation, the depth *z*_*B*_ can be retrieved by evaluating the spectral modulation frequency 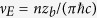 in the reflected spectral intensity, which can be measured by an XUV spectrometer. Here, the refractive index *n* of the material between the top and the buried layer changes the optical path and must be considered. Accordingly, for retrieving the depth of the buried layer with high precision, the dispersion of the host material must be known and included in the analysis (see Methods Summary - Dispersion correction).

## Results

The results of a depth reconstruction in the silicon and water window are shown in Fig. 3. The investigated samples consist of one-dimensional layers of nanometer thicknesses of materials with high refractive index contrast. The samples were produced by sputtering layers onto silicon wafers. The upper panel shows the XCT signal in the silicon transmission window of two 5 nm gold layer separated and buried by silicon layers. For this measurement the XUV spectrometer (spectral-domain-XCT) was used. The slit for spatial filtering was set to a closed position (see also Supplementary Fig. S1 online) to increase spectral resolution. In the reconstructed depth profile the gold layers are clearly separated from each other. Thus, the axial resolution is better than 18 nm. The third peak in the XCT signals is a ghost peak and appears at the depth value of the difference between the two real depths (see Methods Summary - Auto-correlation artifacts).

**Figure 1:**
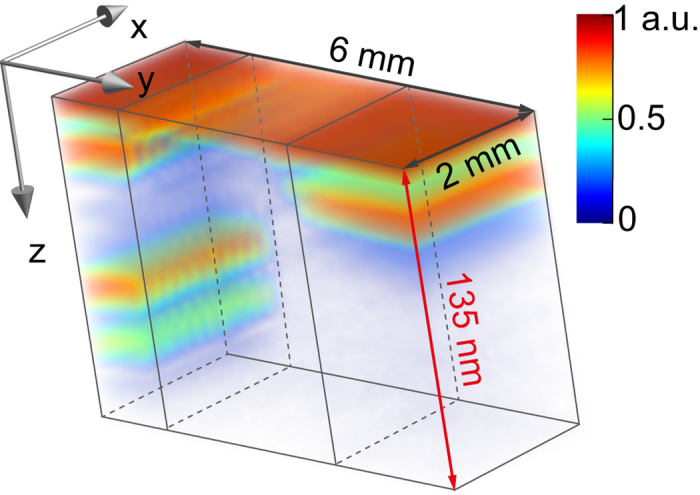
Three-dimensional XCT image of a silicon-based sample with different buried layers of gold. The nano-structures are clearly resolved inside the silicon host material (see also Fig. 4). The depth structure was reconstructed by analyzing the spectral interferogram. Due to the short coherence length of the applied XUV radiation, the axial resolution is better than 18 nm. Lateral imaging was achieved by scanning the focused XUV beam over the sample with a lateral resolution of 200 *μ*m. This can be improved by using spatially coherent XUV sources and XUV optics of highest quality.

In Fig. 4 a more detailed illustration of the tomographic measurement in the silicon transmission window (that was already pictured in Fig. 1) is shown. Since our XCT setup uses the top layer reflection as a reference, we are able to image depth structures relative to the surface. A tomogram with a lateral dimension of 2 × 6 mm^2^ having 3 different layer systems was recorded within 2 hours using XUV radiation in the silicon transmission window (see also Supplementary Movie1 online). A single depth profile was retrieved within one second. For improving the scanning speed the highest available photon flux was applied such that spatial filtering was not active. Therefore, in this case the resolution in lateral dimension was about 200 × 300 *μ*m, only. This can be ascribed to the lateral coherence of the synchrotron beam. However, the axial resolution is still determined by the coherence length of the light source in the order of nanometers. The independency of the axial resolution from the lateral resolution can be seen as a key feature of XCT, particularly in regard to the effort necessary to focus XUV radiation down to nanometer dimensions.

**Figure 3:**
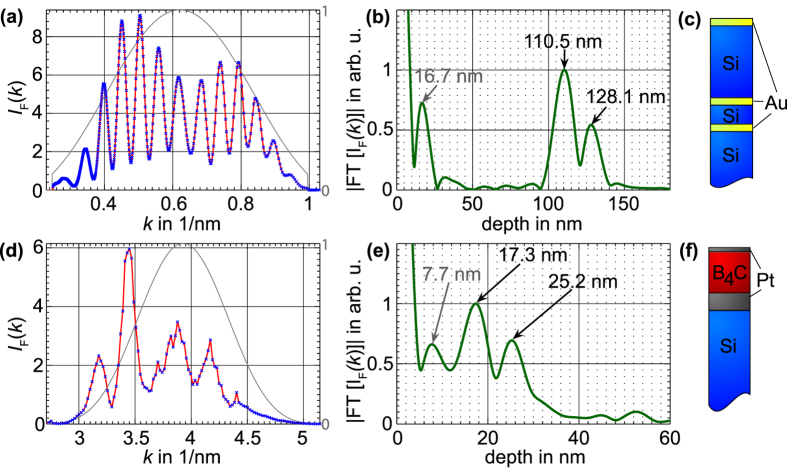
XCT signals in silicon- and water transmission window. (**a**) Recorded reflected spectral intensity in the silicon transmission window from 30–100 eV (40–12 nm) of the layer system (**c**). The blue dots correspond to the CCD-cameras pixels the red curve is the interpolation. The grey curve is the used spectral window for suppression of Fourier artifacts. (**b**) Reconstructed depth profile: The two gold layers appear clearly separated, thus the resolution is better than 18 nm. The first peak at 16.7 nm is a ghost peak and corresponds to the distance between the two real depths. (**d**) Recorded reflected spectral intensity in the water window 280–530 eV (4.4–2.3 nm) of the layer system (**f**). The blue dots correspond to the energy measurement discretization and the red curve is the interpolation. The grey curve is the used spectral window for suppression of Fourier artifacts. (**e**) Reconstructed depth profile: The front and backside of the platinum layer appear separated, thus the resolution is better than 8 nm. The first peak at 7.7 nm is a ghost peak and corresponds to the difference between the two real depths.

**Figure 4:**
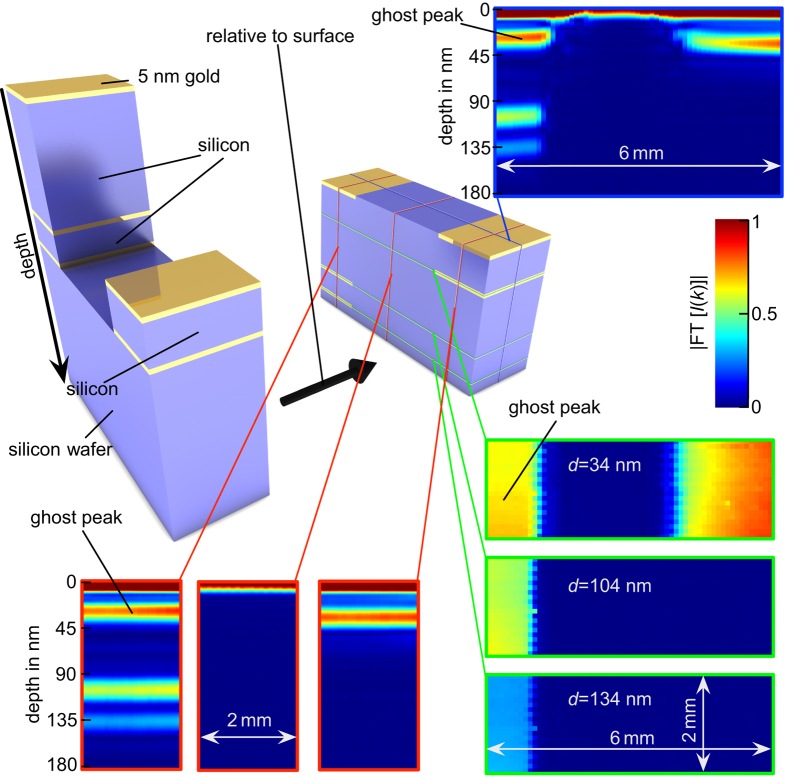
Sketch and cross-sectional images of a volume containing gold structures in a silicon substrate. The **top left** side of the picture shows a schematic sketch of the investigated volume of a nanostructured silicon sample. Due to the fact that XCT exploits the surface reflection as a reference, the sketch in the **middle** of the picture shows the volume how it is expected to be measured. The false color plots in the **right and lower** part of the picture show slices through the volume indicated by the red, green, and blue lines. The plots are retrieved by calculating the Fourier-transform of the measured XUV spectrum for the respective lateral position. Due to the short coherence length of the employed XUV radiation, the axial resolution is better than 18 nm. All buried layers appear clearly visible and separated from each other.

The lower panel of Fig. 3 shows a single XCT scan in the water transmission window of an 8 nm platinum layer buried under a boron carbide layer. Due to lower reflectivity in the water window the signal was too low to achieve a sufficient signal-to-noise ratio with the spectrometer’s grating efficiency and CCD detector noise. Instead, the wavelength of the light source was swept while the signal was recorded with a photo diode (swept-source-XCT) and accordingly the slit was opened fully. The front- and backside of the platinum layer are resolved what corresponds to an axial resolution better than 8 nm. We thus have shown that coherence tomography in the water window is feasible and improved the axial resolution of OCT almost by a factor of thousand.

## Discussion

Due to the simplified setup of the presented first proof-of-principle experiment it is evident that XCT has some disadvantages in its current state. The high reflected capping layer on the sample adds auto-correlation artifacts (see Methods Summary - Auto-correlation artifacts) to the image (ghost peaks). To overcome this limitation one needs to build a complete interferometric setup with a distinct reference arm, which we avoided so far for reasons of simplicity. We already successfully build and tested an XUV interferometer for monochromatic light^17^ and we will adapt it to fit the needs of XCT in the future. Another drawback of the current setup is the lateral resolution of a few hundert micrometers only. The lateral resolution is limited by the source size, its spatial coherence properties, and the focusing optics of the used beamline. The limitations of the current XCT setup can be overcome with more advanced optical components such as multiple toroidal mirrors^18^, Kirkpatrick-Baez Optics^19^, or zone plates^20^. Additionally, a mask with a small hole in front of the sample can be used to limit the area of interaction and thus increasing the lateral resolution at the expense of the usable photon flux. Although the lateral resolution of the current setup is limited, the investigation of deviations of layer thicknesses on a micrometer lateral scale is possible. Thus, samples like multilayer optics or solar cells can be inspected even with the current setup.

Compared to competing nanometer imaging techniques such as soft x-ray microscopy^21^, x-ray diffraction imaging^22^,^23^, and x-ray holography^24^, which utilize XUV and soft x-ray radiation, XCT has the decisive advantage that a broad bandwidth can be exploited efficiently and the depth profile can be obtained quickly. Moreover, the nondestructive reconstruction of the depth profile of a nanostructure providing lateral resolution at the same time is a unique feature of XCT. A comparison of XCT to other soft x-ray imaging techniques is given in the Supplementary Notes online. The ability of XCT to nondestructively produce tomographic images of silicon-based samples with nanometer axial resolution holds promise for applications in the growing sector of semiconductor metrology. Especially, the investigation of lithographic masks could be a promising application^25^. We expect that XCT can become a widely applied microscopy technique, in particular when more prevalent XUV sources like high harmonic generation in gases^9^,^10^ or radiation from laser plasmas^26^,^27^ are used.

## Methods

### XUV sources

We used the undulator beamline BW3^28^ of the DORIS III synchrotron at the DESY facility (Deutsches Elektronensynchrotron) and the undulator beamline UE-112-PGM1^29^ at the BESSY II storage ring (Berliner Elektronenspeicherring-Gesellschaft für Synchrotronstrahlung). To achieve a sufficient spectral resolution it was necessary to use an entrance slit at the position of the beamline focus. Accordingly, the usable photon flux on the sample was limited by the losses at the entrance slit and the reflectivity of the first toroidal mirror. We estimate the photon flux on the sample to be in the range of 10^9^–10^11^ photons per second in 0.1% bandwidth.

### Dispersion correction

In general, the refractive index is a function of the photon energy *n*(*E*), such that the optical path between the layers and so the retrieved depths distinguish even for different photon energies. Furthermore, the angle of incidence in the setup as well as the presence of arbitrary layer combinations has to be considered. Fortunately, the refractive indices of most of the materials in the XUV regime is comparable and the knowledge of the dispersion behavior *n*_*D*_(*E*) of the dominant material^13^ in the sample is sufficient to reconstruct an adequate depth profile. To this end, a transformation of the measured spectral intensity *I*(*E*) from photon energies into spatial frequencies 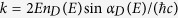 is necessary and includes both, dispersion and the law of refraction (*α*_*D*_(*E*) is the energy dependent angle of the XUV beam in the dominant material to the surface). The depth signal *S*(*z*) is then easily retrieved by applying a Fourier transform 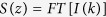 to the spectrum. A more detailed analysis can be found in the Supplementary Notes online.

### Auto-correlation artifacts

When making the transition to many buried layers *z*^*i*^, the interferences and spectral modulations become more complex. Indeed, each optical path length from the top layer to the buried layers gives rise to a well-defined spectral modulation frequency 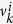 which in turn can be reassigned to a structure at a depth *z*^*i*^. However, the scattered waves from two buried layers *i* and *j* with a distance 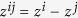 also interfere and generate a characteristic modulation frequency 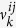. Consequently, the reconstructed depth profiles *S*(*z*) also show a structure at the interspace distances *z*^*ij*^. However, the thin high reflective capping layer on our samples, which acts as a kind of reference mirror, increases the amplitude of the real depth modulation frequencies, such that the amplitudes of the ghost depths are strongly attenuated. The ghost peaks *z*^*ij*^ can also be regarded as relative depth measurements of the structures at *z*^*i*^ and *z*^*j*^.

## Additional Information

**How to cite this article**: Fuchs, S. *et al*. Nanometer resolution optical coherence tomography using broad bandwidth XUV and soft x-ray radiation. *Sci. Rep.* **6**, 20658; doi: 10.1038/srep20658 (2016).

## Supplementary Material

Supplementary Information

Supplementary Information

## Figures and Tables

**Figure 2:**
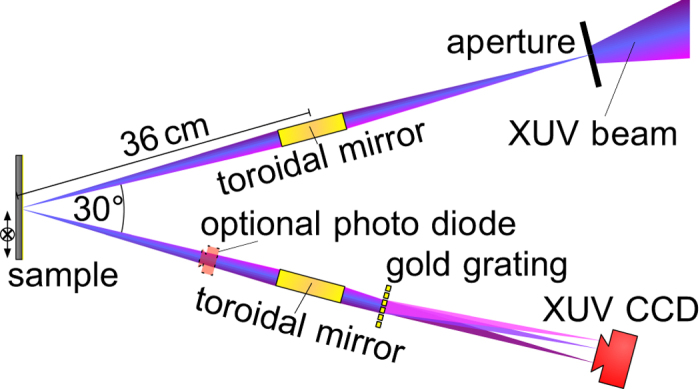
Implementations of XCT as a common-path frequency-domain OCT scheme. The broadband XUV radiation is focused on the sample using an incidence angle of 15° in respect to the sample’s normal. In this setup, an aperture can be used for spatial filtering and enhancing the lateral resolution at the expense of radiation flux. The scattered radiation is recorded with an efficient spectrometer consisting of a focusing mirror, a transmission grating (1000 lines/mm) and a CCD (Back-illuminated XUV-CCD, pixel size 13.5 *μ*m, 2048 × 512 pixels). When the wavelength of the incident XUV radiation is swept, the spectrometer can be replaced by a photo diode as a detector.
