# Supplementary material for: Nanometer resolution optical coherence tomography using broad bandwidth XUV and soft x-ray radiation
Source: Sci Rep. 2016 Feb 10;6:20658. doi: 10.1038/srep20658 (PMC4748318; doi:10.1038/srep20658)
Supplement: Supplementary Information [file srep20658-s1.pdf]

Nanometer resolution optical coherence tomography using broad bandwidth XUV and soft x-ray radiation

Silvio Fuchs[1,2,*,+], Christian Rödel[1,3,+], Alexander Blinne[1], Ulf Zastrau[1], Martin Wünsche[1,2], Vinzenz Hilbert[1], Leif Glaser[4], Jens Viefhaus[4], Eugene Frumker[5,], Paul Corkum[5], Eckhart Förster[1,2], and Gerhard G. Paulus[1,2]**

[1]Institute of Optics and Quantum Electronics, Friedrich-Schiller-University Jena, Jena, Germany
[2]Helmholtz-Institute Jena, Jena, Germany
[3]SLAC National Accelerator Laboratory, Menlo Park, United States
[4]Deutsches Elektronen-Synchrotron DESY, Hamburg, Germany
[5]Joint Attosecond Science Lab, National Research Council of Canada, Ottawa, Canada
[*]silvio.fuchs@uni-jena.de
[**]current address: Ben-Gurion University, Beer-Sheva 84105, Israel
[+]these authors contributed equally to this work

Supplementary Notes

XUV focus

Figure S1 shows the focal spot for different slit widths. The 3D measurement was recorded with an almost fully opened slit and thus the lateral resolution was better than 200 μm x 300 μm.

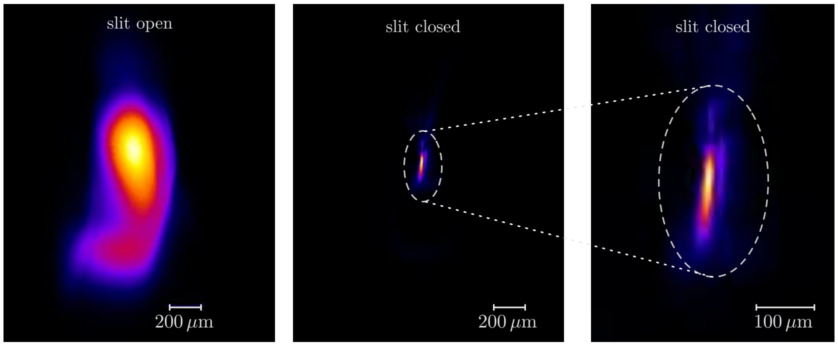

Supplementary Figure S1. Focal spot of the synchrotron radiation on the sample at the undulator beamline BW3: The slit is fully opened in the left picture and almost closed on the right pictures. The 3D measurement was recorded with an almost fully opened slit and thus the lateral resolution was better than 200 μm x 300 μm.

XUV beam splitter

Building a typical OCT setup based on a Michelson interferometer would encounter some crucial technical hurdles in the XUV regime. Due to the short wavelength, the mechanical vibrations of the interferometer arms with respect to each other should be stabilized to the sub-nanometer scale, which would be difficult to realize. Furthermore, an XUV beam splitter with a broad spectral transfer function, a sufficient transmission rate, and a flat surface would be necessary to enable XCT. To construct such a beam splitter would also be a highly demanding technical challenge[17,S1]. Nevertheless, it will be one of the next developments to improve the XCT setup.

Optical properties in the XUV range

For the measurement of the spectral behavior of the signal either an imaging transmission grating spectrometer[14,15] together with a broadband XUV source (spectral-domain XCT) or an XUV-photodiode together with a monochromatic tunable source

(swept-source XCT) were used. The usable bandwidth and therefore the possible resolution of XCT depend on absorption edges of the sample materials. Only in a spectral region with low absorption, i.e. areas between two absorption edges, it is possible to achieve sufficient penetration depths on the order of 1 μm. As mentioned in the article, we chose the silicon transmission window between 12 and 41 nm for silicon-based samples and the water transmission window between 2.4 and 4.4 nm for carbon- and oxygen-based samples to investigate the capability of XCT regarding possible applications in the chip industry and in imaging in the water window.

Data analysis and dispersion correction

In order to include the dispersion of the sample in the analysis, the phase differences $\delta_{i+1} - \delta_i$ of the reflected beams from different layers including their dependence on the refractive index and angle of incidence have to be taken into account. The phase difference between the beams from two layers at depths z_i and z_{i+1} with n_i as the index of the material between these layers, the angles of incidence α_{i-1} and α_i, and the optical frequency ω as indicated in Fig. S2 is

$$\Delta\varphi_{i+1,i} = \omega(\delta_{i+1} - \delta_i) = \frac{2n_i\omega(z_{i+1} - z_i)}{c\sin\alpha_i} - \frac{n_{i-1}\omega l_0}{c}\,. \tag{1}$$

With $l_0 = 2(z_{i+1} - z_i)\cot\alpha_i\cos\alpha_{i-1}$ and the law of refraction $n_{i-1}\cos\alpha_{i-1} = n_i\cos\alpha_i$, the phase difference is

$$\Delta\varphi_{i+1,i} = \omega(\delta_{i+1} - \delta_i) = \frac{2n_i\omega(z_{i+1} - z_i)\sin\alpha_i}{c}\,. \tag{2}$$

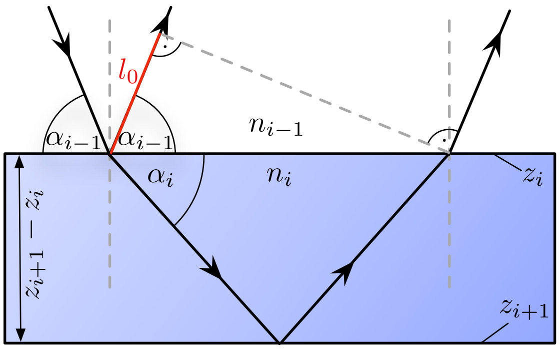

Supplementary Figure S2. Beam path and optical path difference of a single layer.

If several layers are included, the total phase difference is given by the sum ($i > j \geq 1$) in which k_m is the geometric wave number:

$$\Delta\varphi_{i,j} = \omega(\delta_i - \delta_j) = \sum_{m=j}^{i-1} \frac{2n_m\omega(z_{m+1} - z_m)\sin\alpha_m}{c} = \sum_{m=j}^{i-1} k_m(z_{m+1} - z_m) \tag{3}$$

According to the OCT model[2,11], the frequency dependent spectrum at the detector is thus given by

$$S_A(\omega) = S_0(\omega)\left\{R_{\text{ges}}(\omega) + 2\sum_{i>j=1}^{N}\sqrt{R_i(\omega)R_j(\omega)} \times \cos\left[\omega\sum_{m=j}^{i-1}\frac{2n_m(\omega)(z_{m+1} - z_m)\sin\alpha_m(\omega)}{c}\right]\right\} \tag{4}$$

where $S_0(\omega)$ is the spectrum of the light source and $R_{\text{tot}}(\omega) = \sum_{i=1}^{N} R_i(\omega)$ is the total reflectivity. $R_i(\omega)$ is the reflectivity of the i-th layer, which includes the absorption from all layers before:

$$R_{i+1}(\omega) = R_{i+1}^{\text{iso}}(\omega)\prod_{j=1}^{i}(1 - R_j^{\text{iso}}(\omega))^2 \cdot e^{-2n_j''(\omega)(z_{j+1} - z_j)}, \qquad R_1(\omega) = R_1^{\text{iso}}(\omega) \tag{5}$$

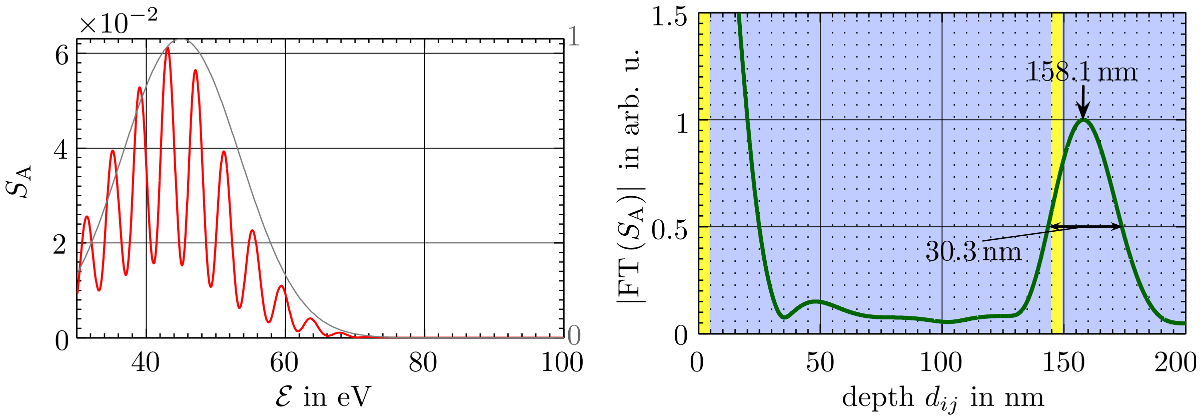

Supplementary Figure S3. Influence of dispersion correction on the XCT signal of a simulated spectrum with the layers 5 nm gold (yellow), 140 nm silicon (blue), and 5 nm gold on a silicon wafer): **(Left)** reflected spectrum (red) and Gaussian shaped spectrum of the light source with a FWHM of 20 eV centered at 45 eV (grey) **(Right)** FT without any corrections ($d_{ij} = z_i - z_j$); the reconstructed depth is bigger than the real depth because dispersion is neglected and the refractive index is assumed to be $n = 1$.

Here, $R_i^{\text{iso}}(\omega)$ is the absolute reflectivity of the isolated i-th layer and $n''_j(\omega)$ is the imaginary part of the refractive index of the j-th layer. In Fig. S3 a simulated reflected spectrum with a Gaussian shaped light source and its reconstruction is plotted. The influence of dispersion on the resolution of XCT can easily be seen if one considers the reflected spectrum of two layers. Thus, the reflected spectrum is

$$S_A(\omega) = S_0(\omega)\left\{R_{\text{ges}}(\omega) + 2\sqrt{R_1(\omega)R_2(\omega)}\cos\left[\omega\frac{2n_1(\omega)(z_2 - z_1)\sin\alpha_1(\omega)}{c}\right]\right\}. \tag{6}$$

In order to reconstruct the depth profile a Fourier transform (FT) is necessary. This leads to

$$\begin{aligned} \text{FT}\left[S_A(\omega)\right] &= \int_{-\infty}^{\infty} S_A(\omega)e^{-i\omega\tau}\,\mathrm{d}\omega \\ &= \text{FT}\left[R_{\text{tot}}(\omega)\right]\otimes\text{FT}\left[S_0(\omega)\right] + 2\text{FT}\left[\sqrt{R_1(\omega)R_2(\omega)}\right] \\ &\otimes\left\{\text{FT}\left[S_0(\omega)\right]\otimes\text{FT}\left[\cos\left(\omega\frac{2n_1(\omega)(z_2 - z_1)\sin\alpha_1(\omega)}{c}\right)\right]\right\} \end{aligned} \tag{7}$$

The influence of dispersion is now pretty obvious. The structural information is encoded in the phase of the cosine function $(z_2 - z_1)$, whereas the modulation frequency is "chirped" by the refractive index $n_1(\omega)$ which in general will lower the resolution. In addition, the FT of the cosine function is broadened by the convolution $\otimes$ of the FT of the reflectivity (wich includes absorption) and of course the FT of the source spectrum, which defines the resolution limit, i.e. the coherence length. If no information about the sample material is known the influence of dispersion which lowers the axial resolution cannot be compensated. Fortunately, at least the dominant material of the samples such as silicon in semiconductor industries will be known. This information can be included into the reconstruction analysis to improve the resolution and the absolute accuracy of the method.

In the first step of the analysis, we normalized our measured spectra with a reference spectrum recorded on a blank silicon surface. This reference signal includes the spectrum of the light source as well as any other influences of the detection system such as grating efficiency and toroidal mirror reflectivity. By calibrating the measured data with these references, the spectrometer doesn't need to be calibrated in terms of efficiency. Additionally, the correction leads to an effective top-hat shaped spectral illumination with the borders ω_{min} und ω_{max} and thus to the shortest possible coherence length, i.e. best resolution.

$$\Theta(\omega, \omega_{\text{min}}, \omega_{\text{max}}) = \begin{cases} 1 & \text{for } \omega_{\text{min}} \leq \omega \leq \omega_{\text{max}} \\ 0 & \text{else} \end{cases}. \tag{8}$$

Certainly, this is only possible if the dynamic range of the detector is high enough to detect sufficient signal in the entire spectral window with the available source spectrum. The such corrected spectrum corresponds to the reflectivity $R_A(\omega)$ of the sample.

The influence of this correction is shown in Fig. S4.

$$R_{\mathrm{A}}(\omega) = \frac{S_{\mathrm{A}}(\omega)}{S_0(\omega)} = \Theta(\omega,\omega_{\min},\omega_{\max})\Bigg\{R_{\mathrm{tot}}(\omega) + 2\sum_{i>j=1}^{N}\sqrt{R_i(\omega)R_j(\omega)} \times \cos\left[\omega\sum_{m=j}^{i-1}\frac{2n_m(\omega)(z_{m+1}-z_m)\sin\alpha_m(\omega)}{c}\right]\Bigg\} \tag{9}$$

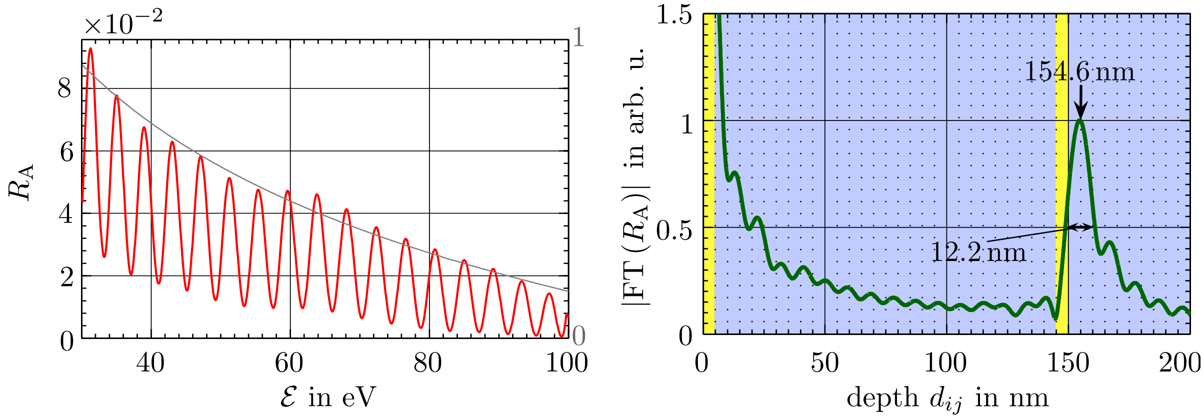

Supplementary Figure S4. **Influence of dispersion correction on the XCT signal** for the same sample as in fig. 7: **(Left)** reflected spectrum normalized with the spectrum of the light source (red) and rough trend of the overall reflectivity of the sample (grey) **(Right)** FT of the corrected spectrum, still without dispersion correction ($d_{ij} = z_i - z_j$); The resolution is improved.

Since the trend of the dispersion and thus the reflectivity and absorption of the layers is changing slowly over the spectrum, it is possible to deconvolve it from the measured spectrum. In general, the dispersion contains no significant modulations. Thus, no information about the depth structure, which is encodes in the modulation frequencies, is influenced. Therefore, we corrected the measured spectrum in a second step with the reflectivity $R_{\mathrm{g}}(\omega)$ of an isolated layer of the material which is dominant in the sample, i.e. silicon or boron carbide. This leads to approximately constant reflectivities.

$$R_{i,\mathrm{g}}(\omega) = \frac{R_i(\omega)}{R_{\mathrm{g}}(\omega)} \approx \text{const.} \tag{10}$$

After this step the corrected reflectivity $R_{\mathrm{A,g}}(\omega)$ is

$$R_{\mathrm{A,g}}(\omega) = \frac{R_{\mathrm{A}}(\omega)}{R_{\mathrm{g}}(\omega)} \simeq \Theta(\omega,\omega_{\min},\omega_{\max})\Bigg\{R_{\mathrm{ges,g}} + 2\sum_{i>j=1}^{N}\sqrt{R_{i,\mathrm{g}}R_{j,\mathrm{g}}} \times \cos\left[\omega\sum_{m=j}^{i-1}\frac{2n_m(\omega)(z_{m+1}-z_m)\sin\alpha_m(\omega)}{c}\right]\Bigg\}. \tag{11}$$

and is shown in Fig. S5.
In order to include dispersion and refraction and to simplify the phase term in the cosine function in eq. 11 we assume the refractive index to be the same for all layers. Therefore, we use the refractive index n_{D} of the material which is dominant in the sample because in most of the cases the main material of the sample is known. Note that the refractive index is still a function of ω. In order to do the approximation, it is useful to perform a coordinate transformation to the geometric wave number

$$k_{\mathrm{D}} = k_{\mathrm{D}}(\omega) = \omega\frac{2n_{\mathrm{D}}(\omega)\sin\alpha_{\mathrm{D}}(\omega)}{c}. \tag{12}$$

The corrected spectrum is then

$$R_{\mathrm{A,g}}(k_{\mathrm{D}}) \simeq \Theta(k_{\mathrm{D}},k_{\mathrm{D,min}},k_{\mathrm{D,max}})\left\{R_{\mathrm{ges,g}}(k_{\mathrm{D}}) + 2\sum_{i>j=1}^{N}\sqrt{R_{i,\mathrm{g}}(k_{\mathrm{D}})R_{j,\mathrm{g}}(k_{\mathrm{D}})}\cos\left[k_{\mathrm{D}}(z_i - z_j)\right]\right\} \tag{13}$$

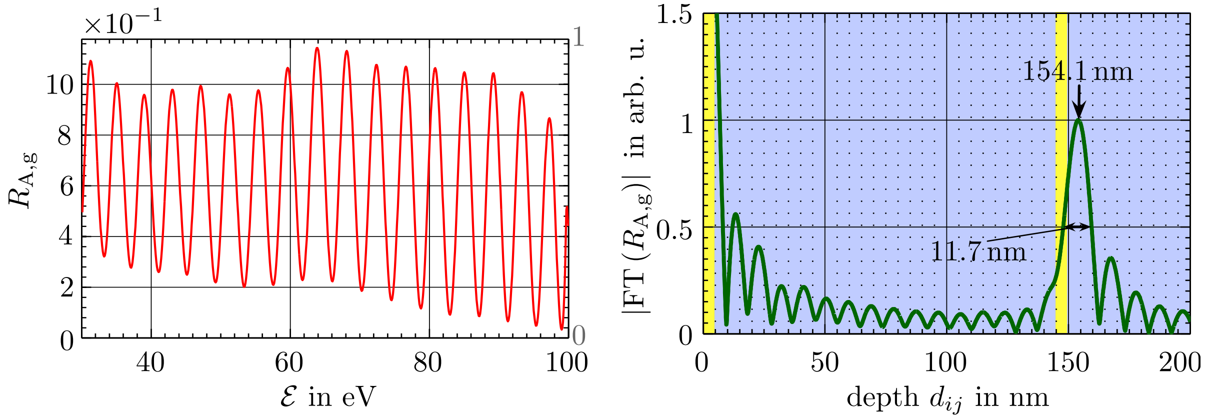

Supplementary Figure S5. Influence of dispersion correction on the XCT signal for the same sample as in fig. 7: **(Left)** reflected spectrum normalized by the spectrum of the light source and deconvolved from the rough trend of the overall reflectivity of the sample (red) **(Right)** FT of the corrected spectrum, still without dispersion correction ($d_{ij} = z_i - z_j$); the resolution is improved further.

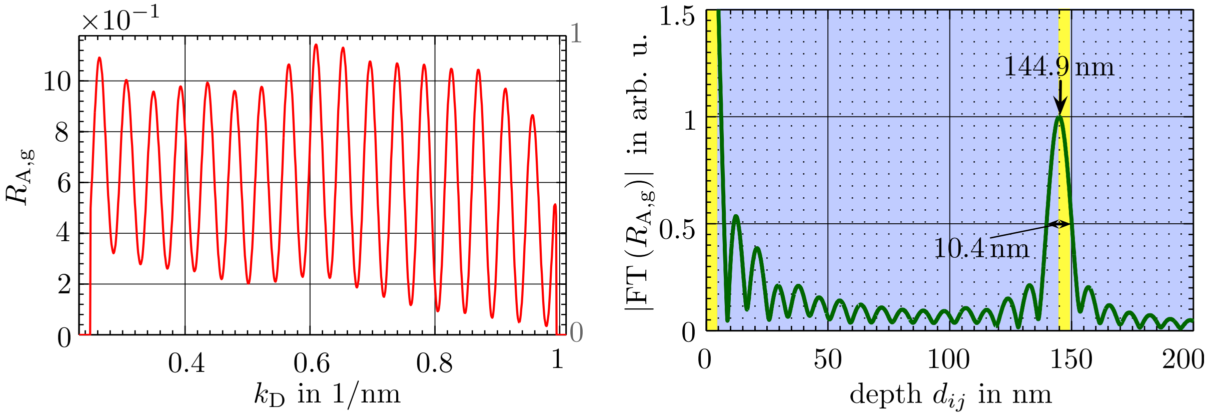

Supplementary Figure S6. Influence of dispersion correction on the XCT signal for the same sample as in fig. 7: **(Left)** reflected spectrum normalized by the spectrum of the light source and deconvolved from rough trend of the overall reflectivity of the sample plotted over the geometric wavenumber k_D with silicon dispersion as dominant material (red) **(Right)** FT in the k_D-space of the corrected spectrum which includes dispersion ($d_{ij} = z_i - z_j$); The resolution is improved further and the reconstructed depth is equal to the real depth.

and is plotted in Fig. S6.
The top-hat shaped spectral window of the corrected spectrum leads to a very high axial resolution but also to very high Fourier artifacts. To suppress these artifacts, we use a Kaiser-Bessel spectral filtering, which is shaped as follows

$$F(k_D) = \begin{cases} \frac{I_0\left[\beta\sqrt{1-\left(\frac{2(k_D-\gamma)}{\eta}\right)^2}\right]}{I_0(\beta)} & \text{for} \quad k_{D,\min} \le k_D \le k_{D,\max} \\ 0 & \text{else} \end{cases} \tag{14}$$

Here, $\eta = k_{D,\max} - k_{D,\min}$ and $\gamma = (k_{D,\max} + k_{D,\min})/2$. I_0 is the modified Bessel function. β is a free parameter that determines the strength of the filtering. The spectrum after all correction steps is then

$$R_{A,g,F}(k_D) = F(k_D) \cdot R_{A,g}(k_D) \tag{15}$$

In order to obtain the depth structure one only needs to perform a FT. The effect of the spectral filtering is shown in Fig. S7.

Comparison to other x-ray imaging and thin film analysis techniques

Several techniques have been developed to investigate nanostructured and layered samples. In the context of layered samples, optical ellipsometry[S2] as well as x-ray reflectometry[S3] is widely used. In fact, monolayers can be measured nondestructively

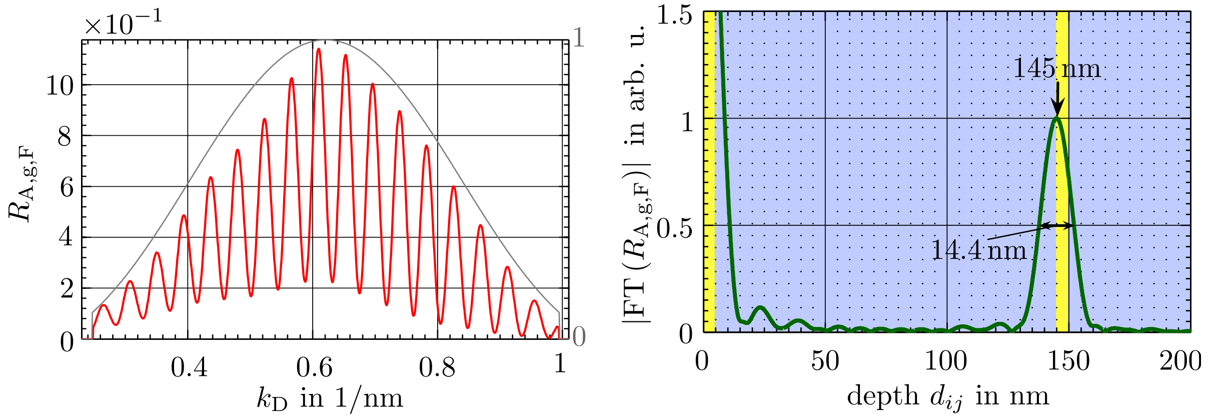

Supplementary Figure S7. Influence of dispersion correction on the XCT signal for the same sample as in fig. 7): **(Left)** reflected spectrum normalized by the spectrum of the light source and deconvolved from the rough trend of the overall reflectivity of the sample plotted over the geometric wavenumber k_D with silicon dispersion as dominant material filtered by a Kaiser-Bessel window (red), Kaiser-Bessel window with $\beta = 4$ (grey) **(Right)** FT in the k_D-space of the corrected spectrum, which includes dispersion ($d_{ij} = z_i - z_j$); The resolution decreases but Fourier artifacts are suppressed.

with high precision. However, these techniques typically cannot retrieve complex samples and provide little lateral resolution. Transmission electron microscopy (TEM)[S4] on the other hand offers extremely high lateral resolution but samples must typically be cut. Consequently, the nanostructure is destroyed and only test samples can be investigated. Moreover, the depth structure is merely retrieved for a slice through the sample. XCT has the big advantage that in principle both high resolution in lateral and axial direction can be obtained without destroying the sample.

In the context of XUV and soft x-ray imaging of more complex samples, a variety of techniques have been demonstrated. For instance, conventional soft x-ray microscopes have been developed using zone plates[21] or multilayer-coated Schwarzschild objectives[S5]. Moreover, nanostructures are investigated by measuring the diffraction pattern of soft x-ray radiation. A review of coherent diffraction imaging can be found in Ref. 22. Methods such as x-ray ptychography[S6] and x-ray holography[24] are based on the technique of coherent diffraction imaging. Despite the large progress that has been achieved in these microscopy techniques in the last years they require a high flux of monochromatic x-rays which are presently only available at large scale synchrotrons and x-ray free-electron lasers. A decisive advantage of XCT is that broadband XUV radiation can be exploited efficiently which is available at many laboratories by using small scale laser systems and high harmonic generation.

For the special application of semiconductor and mask inspection, the introduced techniques are slightly modified. For instance, soft x-ray scatterometry is applied to investigate the critical dimension of periodic structures in lithography masks and gratings[S7]. Moreover, XUV microscopes are used for defect inspection at the surface[25,S8]. Interferometric techniques in the XUV are extremely challenging[S9] but allow for phase defect inspection. XCT could be a decisive step since high axial resolution can be obtained.

6. Sample design The samples were manufactured by the group of Torsten Feigl from the Fraunhofer Institute for Applied Optics and Precision Engineering. They used sputtering techniques and calibrated coating algorithms to produce the layer systems with high accuracy. The lateral layout of a coated silicon wafer with different layer systems and a picture of it are shown in Fig. S8. The black rectangle indicates the area where the 3D measurement was taken.

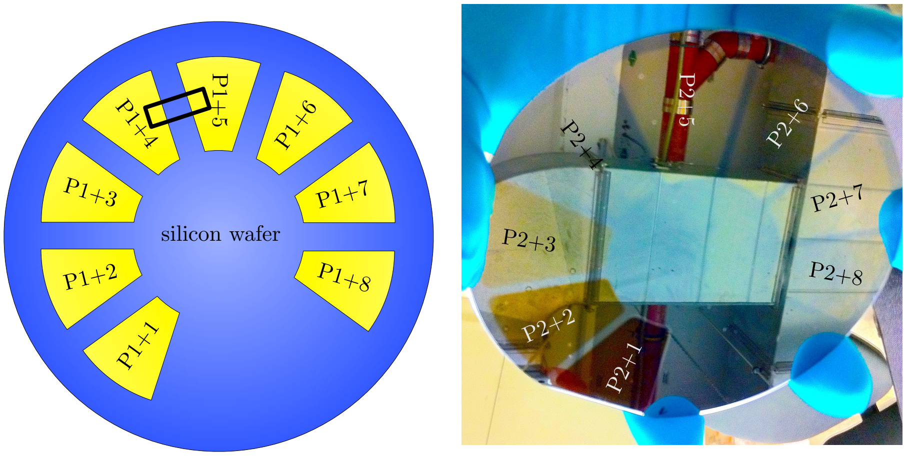

Supplementary Figure S8. Two dimensional design of different layer systems on a silicon wafer: The left graph shows a schematic sketch. The right picture shows a photograph of the used wafer. The black rectangle in the left picture indicates the area which has been observed during the 3D measurement.

Supplementary References

S1. Morlens, A. S. *et al.* Study of XUV beam splitter flatness for use on a Michelson interferometer. *Laser Part. Beams* **22,** 279-284 (2004).

S2. McCrackin, F. L. *et al.* Measurement of Thickness and Refractive Index of Very Thin Films and Optical Properties of Surfaces by Ellipsometry. *J. Res. Natl. Stand. Sec. A* **A 67,** 363-377 (1963).

S3. Chason, E. & Mayer, T. M. Thin film and surface characterization by specular X-ray reflectivity. *Crit. Rev. Solid State Mater. Sci.* **22,** 1-67 (1997).

S4. Petford-Long, A. K. & Chiaramonti, A. N. Transmission Electron Microscopy of Multilayer Thin Films. *Annu. Rev. Mater. Res.* **38,** 559-584 (2008).

S5. Artyukov, I. *et al.* X-ray Schwarzschild objective for the carbon window (λ~45 nm). *Opt. Lett.* **34,** 2930 (2009).

S6. Dierolf, M. *et al.* Ptychographic X-ray computed tomography at the nanoscale. *Nature* **467,** 436-439 (2010).

S7. Gross, H. *et al.* Profile reconstruction in extreme ultraviolet (EUV) scatterometry: modeling and uncertainty estimates. *Meas. Sci. Technol.* **20,** 105102 (2009).

S8. Hamamoto, K. *et al.* Mask defect inspection using an extreme ultraviolet microscope. *J. Vac. Sci. Technol. B Microelectron. Nanom. Struct.* **23,** 2852-2855 (2005).

S9. Haga, T. *et al.* At-wavelength extreme ultraviolet lithography mask inspection using a Mirau interferometric microscope. *J. Vac. Sci. Technol. B Microelectron. Nanom. Struct.* **18,** 2916-2920 (2000).
